# Understanding the Influence of Community-Level Determinants on Children’s Social and Emotional Well-Being: A Systems Science and Participatory Approach

**DOI:** 10.3390/ijerph19105972

**Published:** 2022-05-14

**Authors:** Brenda T. Poon, Chris Atchison, Amanda Kwan

**Affiliations:** 1 School of Population and Public Health, Faculty of Medicine, University of British Columbia, Vancouver, BC V6T 1Z4, Canada; chris.atchison@ubc.ca; 2 Occupational Science and Occupational Therapy, Faculty of Medicine, University of British Columbia, Vancouver, BC V6T 1Z3, Canada; amanda.kwan@ubc.ca

**Keywords:** child development, early childhood, child mental health, child social and emotional well-being, systems science, community-based research, community capital, socio-ecological model, mixed methods, participatory approaches

## Abstract

Healthy social and emotional development and longer-term outcomes for children are shaped by factors across the multiple levels (micro, meso, exo, macro) of a child’s environment. By employing a novel systems science and participatory approach, we were able to co-produce a series of causal loop diagrams that detail the complex relationships between variables operating at the community or neighborhood environment level (e.g., features of the built environment such as: housing type, access, availability, and location; parks and greenspace, facilities such as community services, and other service infrastructure such as transit), and highlight the individual and collective impacts these relationships can have on the subsystem surrounding a child’s social and emotional well-being. Our approach provides a unique lens of knowledge through which communities can identify key leverage points for action and (re)design of community spaces, practices, and policy.

## 1. Introduction

In Canada, more than one-third of girls and one-quarter of boys in Grade 6 reported high levels of emotional problems, such as depression, sadness, anxiety, and sleeping issues [1]. The situation does not improve by Grade 10, with 38% of girls and 24% of boys reporting feeling depressed or ‘low’ at least once a week. Vulnerabilities in social and emotional development are not only prevalent in middle and secondary school-age populations, but are also seen in early childhood, where reported challenges in social competence and emotional maturity are rising. Research suggests that mental health concerns often begin exhibiting symptoms during childhood [2]. Alarmingly, children’s experiences of social, emotional, and behavioral challenges can have lifelong impacts on their quality of life and overall well-being. For example, they may contribute to difficulties with educational attainment [3] increased likelihood of engagement in physical violence and illegal behavior, and substance abuse in adolescence and adulthood [4].

The developmental status of kindergarten children in British Columbia (BC), Canada shows a rising population-level trend in overall vulnerability, with an increase of 2.3% over the last 12 years to 32.2%. The greatest contributors to this elevated vulnerability are low emotional maturity (4.2%) and social competence (2.4%) scores [5]. These domains are in fact interrelated, with developmental outcomes relating to both mental and physical well-being [6]. For example, children who are emotionally distressed may have difficulties learning in school because they are preoccupied, have trouble paying attention, and find it difficult to remember what is taught in the classroom [7,8,9]. Children who experience difficulties in school are less likely to complete high school, which in westernized societies is deemed a necessary precondition for the pursuit of higher learning and, in today’s economy, for stable, well-paid employment [10]. An estimated 50–70% of adult mental health disorders have roots in adolescence, with strong associations to childhood social and emotional (SE) vulnerability. 

Influences both proximal (i.e., downstream determinants) and distal (i.e., upstream determinants) to the child are recognized as highly impactful determinants of health [11]. Proximal influences such as physical health and disease, parenting capacity and stress, and adverse childhood experiences/exposures have lifelong impacts on children’s health and well-being, with outcomes exacerbated by subsequent risks of mental disorders, social disintegration, and violence [12]. Distal influences such as the impacts of poor living conditions and regular exposures to discrimination or a violent community may “get under the skin” by influencing proximal factors that directly impact children’s health and well-being [13,14].

Increasingly, child development studies have used ecological systems theory [15,16,17,18] to identify the influences that lie beyond the individual. Results from such studies have illustrated that children’s social and emotional development is influenced by multiple environments, from the most intimate or micro-level settings of the family dynamic (e.g., [19,20,21,22,23], to the meso-level qualities of the community services, programs they attend, and characteristics of the neighborhoods where they live and play, to the exo-level policy environments that guide the functioning of the local or community institutions designed to support them, to the broad macro-level structural or cultural products such as policies, social norms and values, and belief systems that shape and are shaped by different environments that influence child development (e.g., [24,25]). Despite increasing recognition of this multi-level system, the complex pathways that connect distal and proximal factors across various micro-, meso-, exo-, and macro-levels and the impacts these have on child well-being are not well understood and merit further empirical attention [14]. 

Notwithstanding the wealth of knowledge gained about prevention efforts, which have contributed greatly to understanding parent-child relationships, parenting practices, and parent mental health [26,27], and programs that target specific types of disorders or high-risk groups, there has been a call for increased empirical attention to distal influences on children’s health and well-being [14]. In a priority-setting exercise for the WHO in 2015 regarding primary concerns for research on early child development, the most prominent research questions among 74 international experts in child development emphasized focusing on enabling environments at a community level that support families to nurture and care for their young children, including the need for enhanced knowledge about localized or community-level services and supports for children and their parents, training of health workers and non-specialists to deliver early childhood interventions effectively, and interventions at a systems level that require greater coordination and integration of existing community and neighborhood service delivery platforms [28].

Increasingly, community-based systems science has been used as a promising approach for gaining insights about key drivers of health in cities [29,30,31]. This approach involves active participation of stakeholders to identify public issues and multiple interacting factors impacting a particular problem or issue experienced within the same community, and also helps build local collective capacity for understanding and responding to these issues [32]. The opportunity to employ innovative methodological and analytic techniques, specifically those afforded by the implementation of a community-based participatory systems science approach for exploring community-level or neighborhood environments in which young children live, has important implications for better understanding neighborhood-effects and childhood-development research, and the ability to meaningfully inform policy. Accordingly, the aim of our study was to apply a community-based systems science approach to understand the complexity of factors that operate at and beyond a community level to impact the social and emotional health and well-being of children under 5 years old in one city in interior British Columbia, Canada.

## 2. Materials and Methods

### 2.1. Participants 

Eligibility for participation was intentionally kept broad to include anyone working in an area related to children’s development and/or social and emotional well-being within our partner community of Kamloops, British Columbia (BC). Our recruitment strategy was to engage a diverse group of individuals representative of a range of sectors that influence the environments and opportunities available for children, as well as the practices and policies that govern these spaces. Using a key community contact embedded within the Kamloops network supporting children and families, and employing respondent-driven or network recruitment, we identified a number of central organizations and extended an invitation to participate. We also encouraged individuals who received this initial invitation to extend it to their personal and professional networks, as appropriate. The nature of our recruitment approach made assessing the exact number of potential participants who received invitations to take part in the sessions impossible to accurately determine. Our resulting group of participants included 25 individuals from five areas of work/sectors: childcare, education, service delivery, healthcare, and other. Each of these individuals attended at least one of three community-based group model building (GMB) sessions, with 13 (52%) attending at least two sessions, and seven (28%) attending all three sessions. A breakdown and summary of participants by session is provided in Table 1. The sessions began after review and approval of the study protocol by the University of British Columbia’s Behavioral Research Ethics Board (BREB; on 31 January 2018; UBC BREB no. H17-01850), and informed consent was provided by all participants.

### 2.2. Procedure 

GMB is a systems-based approach for understanding the processes and complexities within a dynamic system. This approach involves a participatory process, which engages stakeholders and community partners in a series of sessions or workshops where a number of pre-determined activities called ‘scripts’ are undertaken [32,33]. Within systems science, causal loop diagrams (CLDs) are frequently used with GMB techniques to qualitatively represent the dynamic influences between factors or variables that are believed to drive system behaviors associated with a particular phenomenon of interest. 

CLDs provide an ability for groups of knowledge co-producers to develop visual indicators of the direction and polarity of relations between key variables identified as being significant for understanding the causal sequences operating within a system, thus allowing for the development of a deeper understanding of how the system functions and changes without actually applying a quantifiable or mathematical value to inter-variable associations [34]. The direction of the relationship between two variables is specified by a line with an arrowhead, while a positive or negative sign attached to the arrow indicates the polarity of the relationship between the variables. When the polarity is the same (+), a variable moves in unison with its proceeding variable. For example, the number of programs increases with an increase in available space. When the polarity of the relationship between variables is opposite (−), a variable moves in the opposite direction as its proceeding variable. For example, parental stress decreases when access to supports increases. Finally, hash marks in the middle of a line connecting two variables represents a time delay for the impact of one variable on the other to be realized. For example, there is frequently a lengthy delay for the impact of increased economic investment in a program or service to be realized by individual program or service users. The logic of directionality, polarity, and time delay are relatively simple to understand within a closed two-variable system. When we examine more complex systems where we encounter a wider and multi-level range of variables, each with their own connection arrows, polarity, and potential time delays contained within multivariable feedback loops, the true power and utility of the CLD begins to emerge. 

Feedback loops are classified as either reinforcing or balancing. Reinforcing loops depict the effect of an increase or decrease in the ensuing variables along a causal pathway resulting from a reinforcing increase or decrease in an initial or antecedent variable, where the impact of change in the ensuing variables is magnified in the same direction of the antecedent variable (i.e., ++ or −−) [35]. Reinforcing feedback loops thus positively or negatively amplify change and produce patterns of exponential growth or decline [36]. Reinforcing loops that emphasize the amplification of positive change in the system represent a virtuous cycle, while those representing the perpetuation of a negative change represent a vicious cycle. Conversely, balancing loops work to counteract change within a system by either limiting growth or slowing the decline of a variable [36]. In a balancing loop, the effect of changes in variables within the loop is to counteract or balance the direction of the change rather than to accelerate it, thereby slowing down the rate of change to push the system towards stability or equilibrium [37]. Understanding not only the causal relationship between individual variables of a system, but also how they collectively exist to create feedback loops allows for a more in depth and accurate understanding of the impact of systems-level determinants. 

Over a 7-month period, we held three facilitated community-based workshops, each lasting for a full day or half-day. Each community-based workshop was a collaborative session that allowed for co-creation of knowledge between academic and community partners, the sharing of ideas and experiences, including feedback on the sessions themselves, and co-creation of CLDs. Although each session had a unique purpose and objective and was guided by particular “scripts,” flexibility was a key consideration in ensuring community partners remained active in the design of and support for each session’s activities [38]. The right to deviate from the script or modify/revise based on the unique needs of the community and their feedback (e.g., focusing more or less time on an activity), was both allowed and encouraged. By encouraging a more participatory approach, we hoped to increase the sense of community trust and ownership of the knowledge and process, increase the accuracy and interpretation of our CLDs and findings, and situate potential interventions or actions within both the most impactful areas and the areas with the greatest likelihood of success. Each of these has been identified as a potential benefit of a community-based participatory approach for health research and addressing complex health problems [39].

### 2.3. Data Analysis 

**Figure 1:**
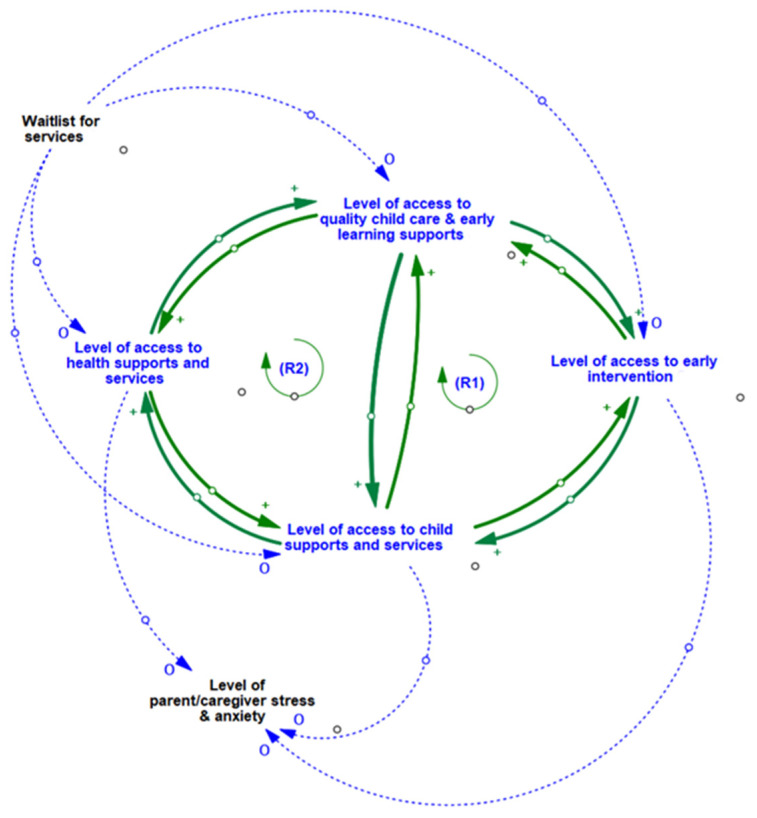
CLD of access points to a range of interconnected community services and program.

**Figure 2:**
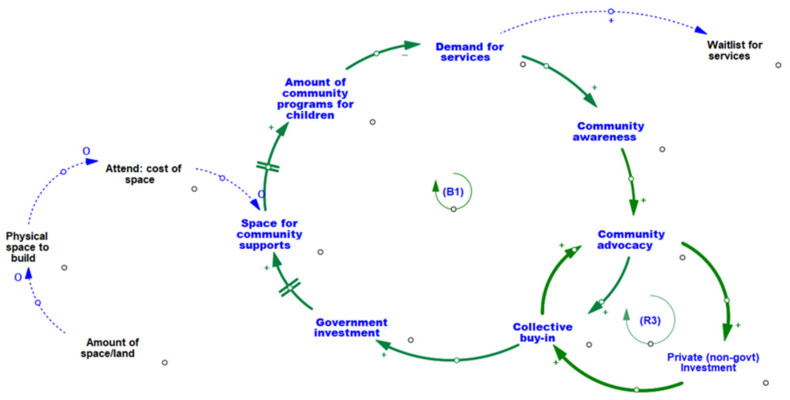
CLD of interrelationships between amount of community programming, levels of collective community action, and availability of physical space and infrastructure.

**Figure 3:**
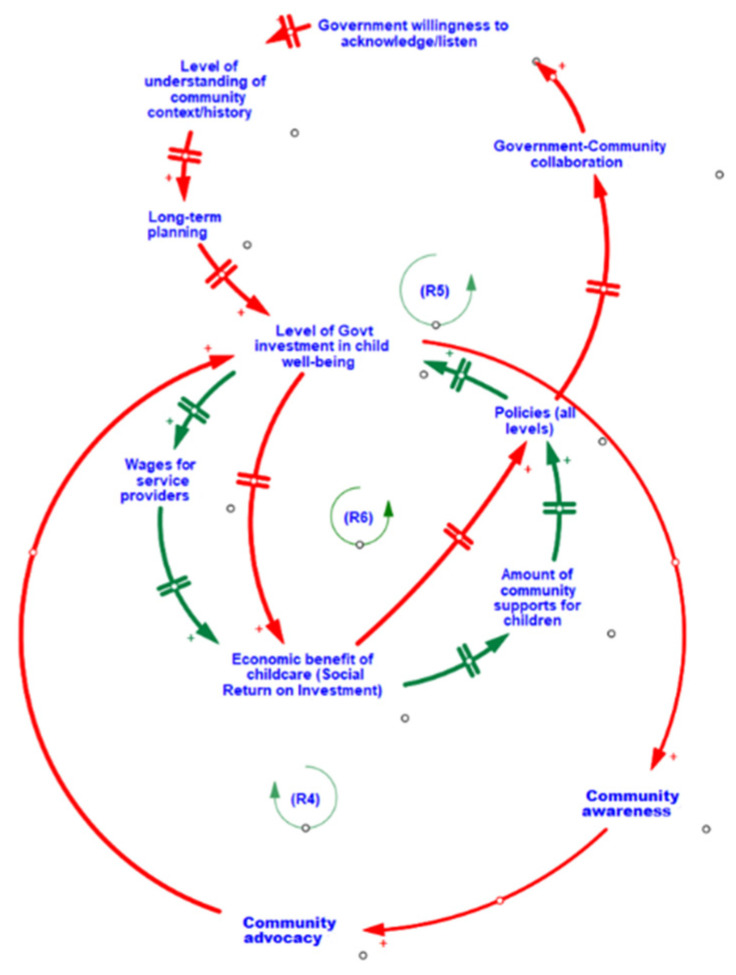
CLD of interrelationships between societal returns on investment, community advocacy, and level of government investment in child well-being.

**Figure 4:**
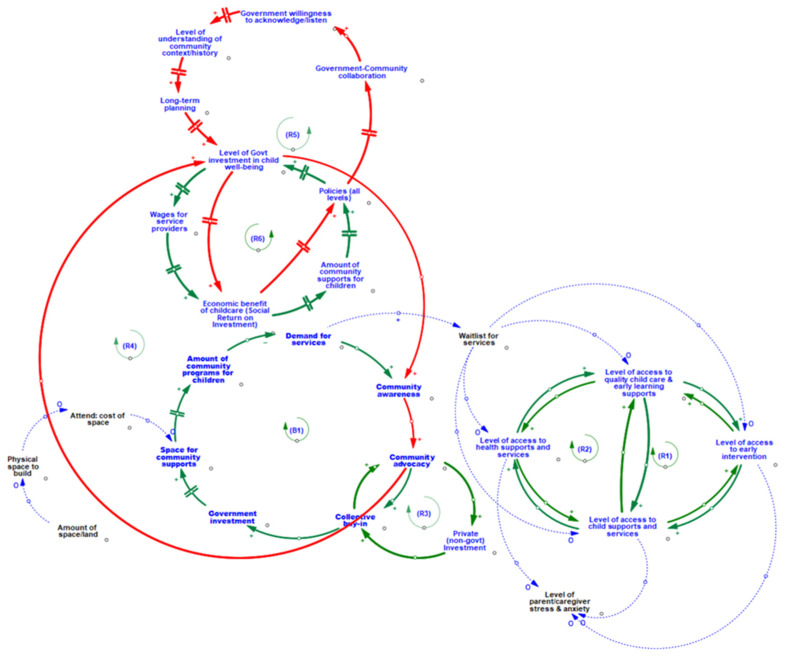
System of community environment influences on children’s social and emotional well-being in Kamloops, BC.

In accordance with the basic tenets of community-based participatory research [40], data analysis involved an ongoing and iterative process that required a collective effort from both the academic research team (ART) and community knowledge co-producers (CKC) who participated in the second and third GMB sessions. Between and after each session, the ART engaged in an extensive process of synthesizing the variables and relations generated from community sessions, developing them into digitally manipulatable versions in Kumu software (https://www.kumu.io, accessed on 8 May 2022), and analyzing them for completeness. Qualitative analysis was also performed on the ‘stories’ developed in session to describe the experiences represented within various CLD pathways. This was an exhaustive process that involved multiple iterations and revisions. The resulting preliminary model was analyzed for accuracy through a guided/facilitated group member checking session directed toward achieving interpretive consensus between the ART and CKC. The final product was a community-driven, co-produced CLD illustrating the system operating with respect to children’s social and emotional well-being within the community (the full CLD can be downloaded at: Supplementary Materials). The Vensim software package, version PLE (Ventana Systems, Harvard, MA) was used to produce visual representations of system and subsystem CLDs, including those presented in Figure 1, Figure 2, Figure 3 and Figure 4 (below).

To enable the creation of a CLD that represents the feedback structure within the community-level or neighborhood environment influences, we first created a detailed map of the entire system. This ensured we were not limited by preconceived notions about what variables exist and which are important at this level. Rather we used the entire systems map to identify major themes at the micro-, meso-, exo-, and macro-levels, and then began to isolate only those individual variables and their associated individual CLDs situated within the community-level or neighborhood environment. For the purposes of our analysis, community-level or neighborhood environment is defined as features of the built environment such as: housing type, access, availability, and location; parks and greenspace, facilities for community programs and services, and other service infrastructure. In this paper, we present and discuss the results from our data collection and analysis that reflect each of these themes and their associated CLDs. We highlight multiple feedback loops as dynamic components of the system surrounding the community-level or neighborhood environment, as well as related (non-looped) variables that are part of the system affecting children’s social and emotional well-being. We then combine these CLDs to display the dynamic interrelationship between several community-level causal sequences.

## 3. Results

Through the group modeling process, participants identified multiple community-level or neighborhood environment variables that have key impacts on children’s social and emotional well-being in Kamloops, BC. In our sessions, the variables at the community-level or in neighborhood environment that participants identified centered primarily on the availability of and access to child and family services and supports. These variables and their interrelationships fell into three key distinct, yet interconnected thematic areas: (1) enabling multiple access points to a range of interconnected community services and programs, (2) increasing the amount of community programming available by strengthening levels of collective community action and ensuring the availability of necessary physical space and infrastructure, and (3) fostering longer-term societal returns on investment and community advocacy through improving government investment in child well-being.

### 3.1. Enabling Multiple Access Points to a Range of Interconnected Community Services and Programs

Two primary feedback loops (R1, R2) influence the relationships in the CLD. The interrelationships between variables represented by Theme 3.1 are reflected in Figure 1, with relevant feedback loops summarized in Table 2. Entry into community services through any one of the four types of community services (i.e., child care and early learning, early intervention, child supports and services, health supports and services) leads to an increased likelihood of accessing each of the other types of community services and programs. Through these key entry points to service access, parents/caregivers are able to receive information about available services and programs through other parents/caregivers and/or service providers, as well as indirect or direct referrals to other types of community services. 

Waitlists directly impact children’s level of access to both (1) health supports and services, and (2) child care and early learning supports. By limiting or delaying access to either of these two points of service entry, waitlists may in turn act to delay or limit access to other types of services available in the community, such as early intervention and other child supports and services. The presence of waitlists for services directly limits parents’/caregivers’ access to a range of services in the community. Parents’/caregivers’ inability to access community-level supports and services leads to increased levels of parent/caregiver stress and anxiety.

### 3.2. Increasing the Amount of Community Programming Available by Strengthening Levels of Collective Community Action and Ensuring the Availability of Necessary Physical Space and Infrastructure

Two primary feedback loops (B1, R3) influence the relationships in the CLD for Theme 3.2 (see Table 2 and Figure 2). The amount of community programming available directly impacts the demand for services from children/families, whereby lesser amounts of programming may translate into fewer programs available to meet families’ needs and higher demands for community programs. When there is a high demand for services from families and community programming is low relative to families’ needs, there is a higher degree of community awareness about the presence of unmet family needs, which in turn may lead to community advocacy efforts to develop collective strategies to address these unmet needs.

The extent that local government supports and invests in these collective community efforts depends on the degree that there is an accumulating cycle of growth and momentum of collective buy-in among community members and strengthening community advocacy efforts, as shown in feedback loop R3. Collective community buy-in can lead to further strengthening of community advocacy efforts, thus creating growth and accumulation of collective momentum towards shared goals and priorities, and further strengthening of community advocacy efforts. 

Strong collective buy-in, created through successful community advocacy efforts and greater local private investment, can also attract the attention and investment of local government to support community efforts, which in turn can be used to locate and fund physical space to accommodate community programming, services, and supports. The amount of programming for children and families in the community is directly impacted by the amount of physical space/built environment available to house the community programs, which in turn is affected by the extent that there is space/land available to accommodate these programs, as well as more generally by the amount of space/land in the community.

### 3.3. Foster Longer-Term Societal Returns on Investment and Community Advocacy through Improving Government Investment in Child Well-Being

Three feedback loops (R4, R5, R6) influence the relationships in the CLD for Theme 3.3 (see Table 2 and Figure 3). The common variable across all three feedback loops is the level of government investment in children’s well-being. The level of government investment leads to a cascade of intersecting reinforcing feedback loops, where there is the potential for continuing growth in government investment that is tied to (1) the strength of community advocacy efforts (R4), (2) the level of the government’s understanding of the local community context and history (R5), and (3) the perceived/actual economic and societal return on investment in supporting early childhood development (R6). 

As feedback loop R4 illustrates, strong community awareness and local grassroots advocacy efforts in childhood well-being can jumpstart collective action, which can, in turn, attract the attention and investment of government in local initiatives. Alternatively, government-led initiatives can have direct impacts on increasing levels of community awareness and advocacy efforts toward specific government priorities. That is, the level of government investment in children’s well-being can have direct impacts on the community’s awareness of key priorities of the government, which in turn can galvanize community collective action towards a shared goal (e.g., cross-organizational collaborative responses to call for proposals from government). Demonstration of successes in community organizing and collective responses to calls for action from the government can lead to further government recognition of a community’s readiness to benefit and successfully achieve beneficial outcomes, and thus may further attract additional future government investment.

In loop R5, the level of government investment in children’s well-being at a community level is impacted by the government’s efforts and willingness to collaborate with the community on local initiatives and to acknowledge, listen, understand, and be responsive to local contexts, histories, perspectives, needs, and priorities. This, in turn, impacts the government’s willingness to engage in long-term planning to collaborate with communities towards shared goals. Loop R6 further highlights the important role of government to support longer-term returns on investment through investments in the human capital required (i.e., adequacy/amount of wages for service providers) to enable greater availability and quality of community supports for children and families. 

The level of government investment in childhood well-being may be dependent on a demonstrated or perceived economic and societal return on investment in young children’s well-being that accrues over time. Enactment of universal childcare, for example, may involve government investment across communities, but this may occur only after consistent evidence of strong returns on investment has accrued over time and a cross-government vision involving multiple intersecting policies has been achieved. Returns on investment in this example may be identified as observed increases to numbers of parents/caregivers who rejoin the workforce earlier and/or the development of healthier and productive adults who successfully contribute to healthy communities and future capital (e.g., human, social, and economic).

### 3.4. Bridging the Multiple Associations and Feedback Mechanisms within the Community Environment 

Thus far, we have presented a number of CLDs that effectively stand alone as ways of describing the thematic dynamics operating within the subsystem of community-level variables. One of the advantages of systems dynamic modeling is that the CLDs and the variables contained within subsystems can be combined to create a more complete and nuanced picture of the situation, thereby improving our ability to understand the relationships between different levels of interaction within the system. Bringing together loops B1, R1, R2, R6, R5, R3, R4, and including additional variable, such as parent/caregiver stress/anxiety, waitlists for services, amount of space/land, physical space to build, and cost of space reveals a CLD that explicates the connections between different thematic areas within the community-level or neighborhood environment subsystem (see Table 2 and Figure 4). 

As we have seen, loops R1 and R2 (Theme 3.1) illustrate two virtuous cycles associated with accessing various health, child care, and early learning supports and services. The first highlights how access to supports and services in the community begets greater access to supports other services, while the second highlights the positive impact that accessing supports and services has on access to early intervention. However, close inspection of this subsystem also reveals a potential for these virtuous cycles to turn vicious if/when accessing supports, services, and/or early interventions is hindered by lengthy waitlists. Under such conditions, the resulting vicious cycle could result in increased levels of parent/caregiver stress and anxiety, which in turn could contribute to exacerbated levels of parent/caregiver-child stress contagion (a known negative influence on child social and emotional well-being).

Similarly, loops R3 and B1 (Theme 3.2) elucidate the interrelationship between balancing and reinforcing processes relating to advocacy, collective buy-in, private and government investment, and the availability (and associated demand for) programs, services, and supports for children within the community. We see that increased collective buy-in, community advocacy, and private investment results in a magnification of positive change (i.e., a virtuous cycle). Community advocacy also contributes to the stabilization or balancing of the system with respect to the cumulative effects of increased community awareness and advocacy, government investment, available physical space, amount of community programs, and resulting reduction in the demand for services. It would appear that this stabilization could have a direct effect of reducing the waitlists for programs and services in the community, which in turn could contribute to a reduction in parent/caregiver-child stress contagion.

Finally, loops R4, R5, and R6 (Theme 3.3) demonstrate three inter-dependent virtuous cycles associated with fostering increased levels of government investment through: (1) increased community awareness and advocacy, (2) the combined influences of wages, social benefits, and support, and (3) social returns, policy, collaboration, and planning. The magnification of positive changes revealed within these three virtuous cycles draws our attention to the central role that government investment in child well-being plays within this subsystem. Additionally, the shared magnification in positive change to the social return on investment from childcare depicted in loops R5 and R6 has a delayed, but direct impact on the amount of community supports for children, creating a vital bridge between the three thematic areas identified in the subsystem of community-level influences on child social and emotional well-being.

## 4. Discussion

Our innovative systems science and participatory approach provides a unique lens of knowledge through which communities can visualize the system operating with respect to children’s social and emotional well-being within the community and identify potential key leverage points for action and (re)design of community spaces, practices, and policy. To our knowledge, this study is the first empirical application of a community-based participatory systems science approach to develop a detailed causal map of the system of primary variables/factors operating within the community that impact children’s social and emotional well-being. Employing this map and the associated CLDs, we were able to visually isolate four individual, yet interconnected subsystems present at the community-level or in neighborhood environment. 

Through the process of creating the CLD represented in Theme 3.1, session participants identified a key reinforcing feedback loop R2, wherein children’s and families’ engagement in one type of community program/service leads to connection with other types of family services and support in the community, thus creating a virtuous cycle where families continue to grow their knowledge and access opportunities. Through review of this causal map with our partner community, we were able to identify that a necessary precursor to creating or sustaining this virtuous cycle is coordination of information and collaboration across the spectrum of services and supports in the community. 

Information sharing about available programs can only occur when each of the programs is well aware of and willing to share information about other available programs. This would require a sufficient level of communication, coordination, and collaboration between providers across different community organizations that is not possible if, instead, community organizations operate in contexts of competition (e.g., for funding or families), isolation, or silos. Our ongoing dialogue with our partner community about the dynamics reflected in this system has led to the identification of a potential leverage point for future community-based research and action that centers on developing strategies to enhance coordination across providers and creating consistent standardized informational resources that could be shared across community organizations, service providers, and families.

The CLD represented in Theme 3.2 contributed to our collective understanding of the dynamic interplay of key tangible and intangible variables in the system influencing young children’s social and emotional well-being. As we expected, tangible components of the system such as the amount of community programs for children are directly linked to other tangibles such as the level of local government investment and availability of physical space/land. It is the intangibles in this system (i.e., levels of community awareness, community advocacy, and collective buy-in), however, that highlight important drivers and cyclic processes that lead to more or less government investment, space for community supports, and community programming for children. When community advocacy efforts are high, for example, there are opportunities for local community members/organizations/businesses to invest financially/non-financially to support community action efforts, and also increased opportunities for relationship building, collaboration, and collective buy-in towards shared goals, which in turn can further strengthen community advocacy efforts. Collective buy-in increases the funding provided by local/private businesses or organizations, which enables communities to allocate new spaces for programs/services and subsequently expand on the availability of programs/services for children and families. The GMB process and resultant CLD provides the community with a valid and reliable means [41] for identifying and interpreting the necessary conditions for and potential impacts of proposed community actions that focus on building community awareness, advocacy, and collective buy-in.

In all three interconnected feedback loops represented in Theme 3.3, session participants identified diverse pathways where the variable level of government investment in child well-being acted as a key driver for levels of community-level advocacy, programs, and supports. Contributing factors for more or less levels of government investment in child well-being in a community were also highlighted (e.g., lack of government understanding of community context and history). The CLD shed light on ways that the level of government investment can operate ‘top-down’ to propel greater community awareness, advocacy, and collective buy-in about government priorities and initiatives, which may in turn lead to additional future government investment. The level of government investment in child well-being at the community level can have direct impacts on increasing the human capital necessary to deliver quality community programs and services for young children and families, which will in time offer economic and societal returns on investment. 

Another interesting nuance of this CLD is the nature of the inter-relationships involving the level of government investment, which primarily operate ‘bottom-up’ and are reflective of the varying extent that community awareness, advocacy, and actions are successful in capturing the attention and interest of decision-makers. One area for future investigation is better understanding of potential leverage points that affect the varying ways that communities use their own community capital (e.g., social, human, built, political, economic) both to facilitate a community’s capacity to collectively identify and address local needs, and to increase likelihood for recognition and selection by decision-makers as an attractive site for government-funded initiatives, as community readiness for action and intervention success in these sites may be deemed high.

There is a considerable body of research detailing the central role that differing forms of capital play in community development [42,43,44,45,46]. It is generally accepted that successful community development requires an inclusive approach that involves all sectors and partnerships among different community stakeholders (e.g., local governments, private business, and community organizations) [47,48,49]. Turner [50] maintains that the operation of three distinct forms of capital—economic, social and political—are particularly vital for successful and sustained community development. Economic (capital) investment is necessary for securing the physical spaces and human capital resources to sustain public and private sector activities, operations, and infrastructure. Social capital—understood as the beneficial normative context arising from social ties among individuals, households, and local institutions within a community [51]—is necessary to ensure that an organized voice of the community, grounded in citizen participation, plays a central role in community-based decision making. Finally, political capital serves to link social and economic capital operation and investments within the community; it reflects the community’s capacity to: collectively set the terms surrounding the negotiation of shared interests, develop a strategy to achieve those interests, and, direct resources that affect the ability of the community to realize goals [52]. 

Drawing upon the information provided from the subsystem of reinforcing and balancing causal loops depicted in Figure 4, it is possible to identify ways that economic, social, and political capital could be mobilized within the community in order to identify potential leverage points relating to the creation and development of community infrastructure that might be used to improve the social and emotional well-being of children. Specifically, Figure 4 illustrates how the parent/caregiver stress and anxiety that is associated with lengthy waitlists and the accompanying restricted access to services could serve as a catalyst for action where parents/caregivers and over-burdened service providers mobilize their collective social capital to generate community buy-in and advocacy (political capital) in order to put pressure on the private (i.e., business) and public (i.e., government) sectors to invest economic capital to fund community initiatives (e.g., acquisition of physical structure, increasing wages, hiring and training of staff/service providers, etc.). If the mobilization of social and political capital within the community is unsuccessful in producing greater levels of private and public sector investment of economic capital desired (i.e., if the result is a vicious cycle), the community would most likely continue to see increased demand for programs and services and the accompanying continued lengthening of waitlists. Such a condition could fuel a recognition that the community needs to intervene in or modify the operation of a particular aspect of the system in order to alter the causal processes currently operating within the community, either toward achieving a magnification of positive changes (i.e., a virtuous cycle) or, at the very least, an outcome of equilibrium (or balancing). The recognition of this necessary condition for change could be the first step in leveraging the understanding of the system facilitated by the CLD in order to affect change.

The emergent themes of our CLD on a community’s built environment and, specifically, the availability of and access to child and family primary care and support services are consistent with previous evidence of key systemic barriers involving critical shortages in specialized services and supports available (e.g., wait lists for mental health services) and challenges in coordination and collaboration of multiple service systems that, individually, may lack the resources and training to attend to children’s social and emotional concerns [53]. Although the individual variables of the CLD in isolation may not necessarily yield new knowledge related to the multi-level social determinants affecting social and emotional well-being, the strengths and contributions of the GMB process and CLDs stem from their capacity to facilitate the development of a more comprehensive understanding of the complex dynamics at play within the system [54], including potential leverage points within the system which can lead to systems change actions over time [55]. They may also help to identify some of the more subtle intangible variables (e.g., collective buy-in) in the system, which may be key precursors for more tangible variables operating within and outside the community, such as government investment.

## 5. Strengths, Limitations, and Future Directions

The group modeling process and the resultant CLDs yielded several important insights that our academic–community partnership can use to guide our future research, with emphasis on the identification of possible leverage points for policy and practice moving forward [35], including key factors for potential system optimization [41]. Our next step is to use the CLDs to pinpoint areas of the system that reflect current community interests and priorities, have the potential to create new knowledge that addresses a community need, and are deemed as feasible and important focal points for guiding future research and community action.

It is important to note that CLDs provide a graphical representation of the key feedback loops and variables operating within the system, but they must be interpreted in the context of the data that informed their construction [36]. The complexity of the resulting systems map is shaped directly by the voices/insights of the knowledge co-producers that are present around the table [54]. In Kamloops, session participants were typically attending based upon their professional roles in community support or service provision. A CLD co-produced by a different group of participants with other types of roles in the community (e.g., all parents/caregivers) may yield a systems map of more or less complexity. An additional challenge, observed in much community-based research, is that the GMB process required considerable participant investment (e.g., community buy-in, time commitment, etc.). This investment may have contributed to some of the participant attrition we observed between the second and third sessions.

GMB of CLDs is part of our current and future research in multiple partner communities across BC, involving groups of varied composition. Ongoing data interpretation of CLDs across communities will further deepen our insights into the key dynamic drivers operating in diverse community or neighborhood environments impacting children’s social and emotional well-being.

## 6. Conclusions

In this paper, we have applied causal loop diagramming techniques from the systems dynamics perspective that we feel are helpful in understanding the key set of drivers affecting the social and emotional well-being of children within the community of Kamloops, British Columbia. Overall, our GMB process integrated a systems science and community-based participatory approach, providing a novel way to enhance collective understanding of the operation of the system and offering the benefit of improving confidence among individual stakeholders in decisions made on the basis of the co-produced knowledge [41]. The individual and combined CLDs we co-produced revealed that multiple community-level variables appear to play important influences on children’s social and emotional well-being within the community. When we narrowed our focus to the variables within the system that were specifically associated with community-level or neighborhood environments, we saw that availability of and access to child and family services and supports were central within this subdomain of the system. Closer inspection revealed three key thematic and inter-related areas: (1) access to interconnected community services and programs, (2) increasing the amount of available community programming through collective community action with respect to the use of physical space and development of infrastructure, and (3) fostering long-term social returns on investment and community efficacy through increased government investment in child well-being. Careful analysis of the model and the specific feedback loops provide valuable opportunities to assess and evaluate what is working or not working to support children’s social and emotional well-being within the community and, importantly, to assess the underlying factors or mechanisms in the system that influence these outcomes. Through continued collective interpretation and updates of the CLD over time, we will be able to continue to highlight potential leverage points that may be used to inform further policy and practice targeting children’s social and emotional well-being at the community level.

## Figures and Tables

**Table 1:** Summary of participants at each group model building session.

Number of Participants in Attendance (%)				
ECD Sector	GMB Session #1	GMB Session #2	GMB Session #3	Total
	(N = 17)	(N = 16)	(N = 13)	(N = 25)
Childcare	4 (23.5%)	3 (18.8%)	1 (7.7%)	4 (16.0%)
Education	3 (17.6%)	3 (18.8%)	4 (30.8%)	4 (16.0%)
Service Delivery	8 (47.1%)	8 (50.0%)	4 (30.8%)	12 (48.0%)
Healthcare	1 (5.9%)	1 (6.3%)	3 (23.1%)	3 (12.0%)
Other	1 (5.9%)	1 (6.3%)	1 (7.7%)	1 (4.0%)

**Table 2:** Summary of key feedback loops in the community/neighborhood environment.

Loop	Loop Name	Description
**R5**	**Shared Understanding and Priorities**	When governments are willing to listen to communities, they are better able to understand the uniqueness and importance of a community’s history and context. This enables more tailored long-term planning and investments towards child well-being, which increases the social return on investment. Increasing returns encourage policies that continue to support child well-being, leading to greater collaborations between community and government, strengthening cross-sectorial relationships and reinforcing the willingness of governments to listen.
**R6**	**The Impact of Government Investments**	When governments increase their investments in child development well-being, the wages for service providers are able to increase, which also increases social return on investment. Increasing social return promotes greater support from the community. Communities with plentiful support for child well-being often engage in community-led initiatives, including community-based research that help to support and guide policies aimed at further promoting child development and well-being. When policies move towards promoting child development and well-being, government investments follow, creating a reinforcing loop.
**R4**	**Community Advocacy and Government**	High levels of community advocacy for child well-being can influence the level of funding received from governments. With increased funding, communities are able to implement new initiatives, which increase community awareness of the state of child vulnerability and importance of the early years. This reinforces the need and presence of community advocacy in support of children’s well-being.
**B1**	**Demand for Program and Services**	When there is high demand for children’s programs/services, long waitlists can exist for families to access supports. Waitlists increase community awareness by being an indicator of unmet community need. With growing awareness comes greater community advocacy and buy-in in support of children and families. Collective buy-in increases the funding provided by local private businesses or organizations, which enable communities to allocate new spaces for programs services (located in built community infrastructure such as community centers), and subsequently expand on existing or create new programs/services for children and families. As more community-located supports become available, the demand and waitlists decrease. This balancing loop illustrates how the system can self-adjust to meet the needs of families with young children.
**R3**	**Advocacy within the Community**	High levels of community advocacy for child well-being can influence the levels of private (non-government) funding. Support from private organizations such as local businesses and banks, contributes to increased awareness and collective buy-in from the community, furthering the level of community advocacy.
**R1**	**Impact of Multiple Access Points to Services/Supports**	Access to one community service access point in the system begets access to additional services and supports. For example, when families with young children are able to acquire better access to health care in the community, their ability to access quality childcare and early learning supports improves, this in turn leads to greater access to early intervention which increases the level of child’s access to family supports, which feed back into improved access to health care. The inter-relationships in this loop are bi-directional. When families with young children are able to acquire better access to health care in the community their ability to access family supports is improved and this increases access to family supports and leads to increased levels of access to early intervention, which in turn enhances access to quality childcare and early learning supports, which contribute to improved access to health care.
**R2**	**Service Access via Connections to Information and Social Networks**	When families with young children are able to access family supports (located in built community infrastructure such as community centers), they gain access to information from staff and other families, which may increase their awareness of other programs and services available within the community (e g., health care, quality daycare) that they may be eligible for. By engaging in these other programs services, families are able to connect with and receive additional family support, creating a reinforcing cycle whereby families continue to grow their knowledge and access opportunities. This creates a virtuous cycle where families who have access continue to gain more access.

## Data Availability

The data presented in this study are openly available and accessible through the above link in Supplementary Materials. For further inquiries or CLD access, please contact the corresponding author.

## Supplementary Materials

The following supporting information (full CLD) can be downloaded at: https://www.mdpi.com/article/10.3390/ijerph19105972/s1.
