# Supplementary material for: Understanding the Influence of Community-Level Determinants on Children’s Social and Emotional Well-Being: A Systems Science and Participatory Approach
Source: Int J Environ Res Public Health. 2022 May 14;19(10):5972. doi: 10.3390/ijerph19105972 (PMC9140710; doi:10.3390/ijerph19105972)

Understanding the influence of community-level determinants on children's social and emotional well-being: A systems science and participatory approach.
Dynamic System Map, Kamloops, BC, Canada

Authors: Poon, Atchison & Kwan.

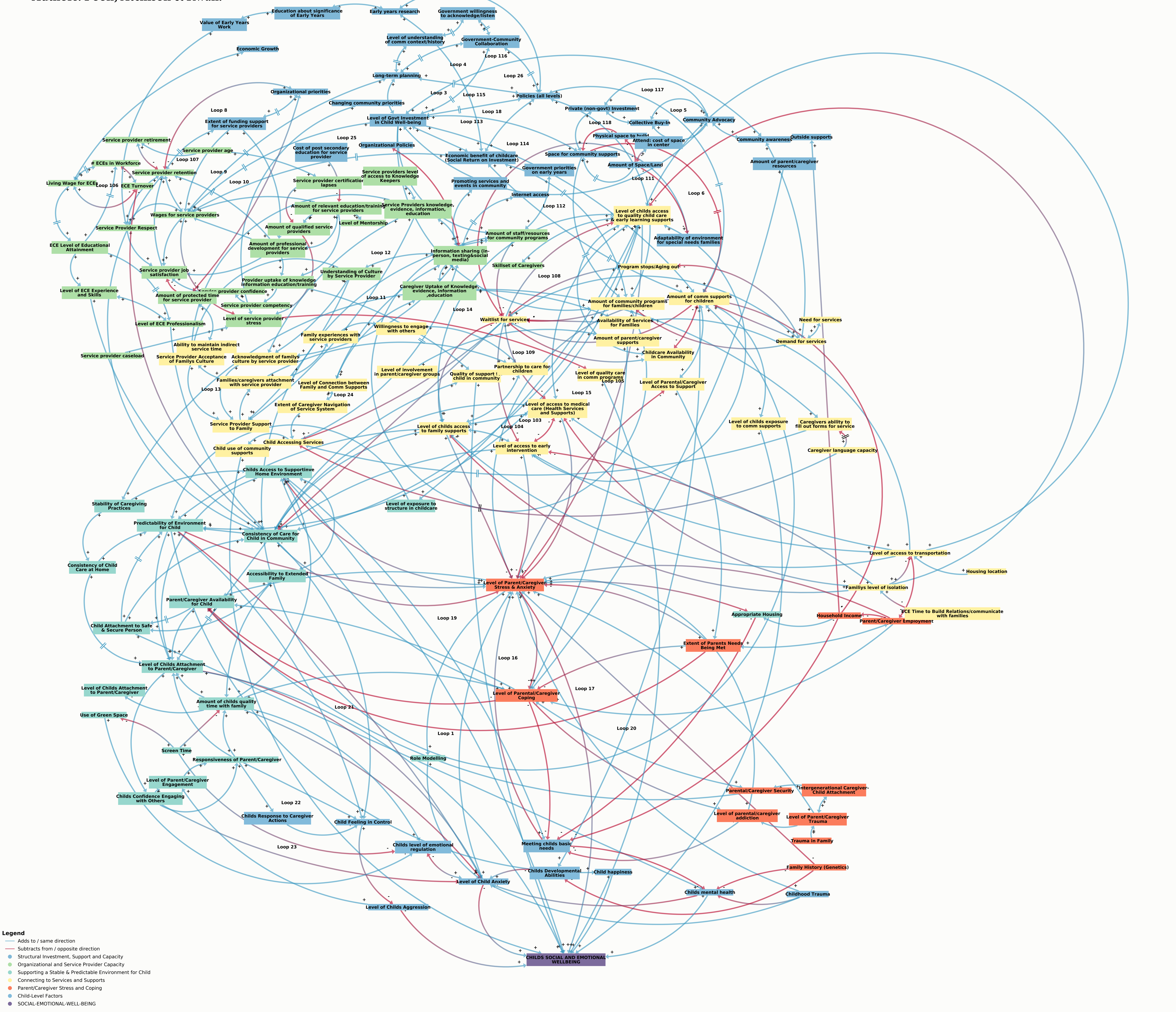

Supplement: Supplementary file 1 [file ijerph-19-05972-s001.zip › ijerph-1690973-supplementary.pdf]
